# Higher hospital level does not improve 30-day survival after road traffic accidents

**DOI:** 10.1038/s41598-025-26519-7

**Published:** 2025-11-21

**Authors:** Viktor Ydenius, Sebastian Djerf, Mats Fredrikson, Robert Larsen, Folke Sjöberg, Attila Frigyesi

**Affiliations:** 1 Department of Biomedical and Clinical Sciences (BVK), Linköping University, Linköping, 581 83 Sweden; 2 Department of Anaesthesiology and Intensive Care, Vrinnevisjukhuset, Norrköping, 601 82 Sweden; 3 Department of Surgery, Ystad Lasarett, Ystad, 271 82 Sweden; 4 Department for Clinical Sciences in Lund (IKVL), Lund University, Lund, 221 85 Sweden; 5 Department of Anaesthesiology and Intensive Care, Linköping University Hospital, Linköping, 581 83 Sweden; 6 Department of Hand Surgery, Plastic Surgery and Burns, Linköping University Hospital, Linköping, 581 83 Sweden; 7 Intensive and Perioperative Care, Skåne University Hospital in Lund, Lund, 221 85 Sweden

**Keywords:** Health care, Risk factors

## Abstract

Globally, road traffic accidents (RTAs) remain a major cause of death, particularly among individuals aged 15–30 years. While Sweden has been at the forefront of traffic safety through the *Vision Zero* initiative, in-hospital management remains crucial in determining RTA outcomes. Drawing on North American evidence suggesting improved survival at trauma centres, the Swedish healthcare system has increasingly emphasised trauma centralisation. However, comprehensive national data from Sweden are scarce. Given the country’s unique demographic and geographic characteristics, including vast sparsely populated areas, direct comparisons with other Western systems are challenging. We analysed the epidemiology and risk factors for 30-day mortality among 95,954 RTA-related hospital admissions in Sweden between 2008 and 2021. Predictors included the ICD-based Injury Severity Score (ICISS), age, sex, Charlson Comorbidity Index (CCI), year of event, and hospital level. Mortality risk was modelled using explainable artificial intelligence (XAI) via Extreme Gradient Boosting (XGBoost) with SHapley Additive exPlanations (SHAP), alongside conventional multivariable logistic regression for comparison. The most influential predictors of 30-day mortality, in descending order, were ICISS, age, CCI, event year, hospital level, and sex. A clear trend toward centralisation was observed, with Level 1 hospitals admitting the most severely injured patients. However, after risk adjustment, the hospital level was not independently associated with 30-day mortality. The XAI model outperformed logistic regression in both discrimination and calibration, confirming these findings. This study represents a comprehensive national analysis of in-hospital outcomes following RTAs in Europe. ICISS, age, sex, and comorbidity influenced mortality risk, while overall survival improved over time. The assumption that trauma centralisation confers a universal survival advantage does not appear to hold in the Swedish context. These findings underscore the need to re-evaluate trauma system design under Scandinavian conditions—ensuring that timely access to hospital care is not compromised by centralisation.

**Supplementary Information:**

The online version contains supplementary material available at 10.1038/s41598-025-26519-7.

## Introduction

Globally, road traffic injuries remain a leading cause of death, particularly among individuals aged 15–30 years. It causes human suffering and a societal burden, costing some countries up to 5% of their gross domestic product annually^1^. Sweden’s national *Vision Zero* initiative—focusing on safer road design, vehicle safety improvements, and evidence-based prevention—has placed the country among the international leaders in reducing traffic-related mortality. Currently, Sweden reports 2.8 deaths per 100,000 inhabitants per year, compared with the European average of 9.3 and the global average of 17.4 per 100,000 inhabitants^2^.

The primary objective of this study is to determine to what extent the observed decline in overall traffic-related mortality is attributable to fewer accidents, fewer injuries, or improved hospital treatment outcomes.

Age, sex, and comorbidity—measured by the Charlson Comorbidity Index (CCI)—are well-established independent determinants of injury outcome^3–5^. For risk adjustment by injury severity, the ICD-based Injury Severity Score (ICISS), developed by Osler et al. in 1996^6^, is regarded as an improvement over the consensus-derived Injury Severity Score (ISS) and is currently considered one of the most accurate methods for trauma risk adjustment^7,8^. ICISS accounts for all of a patient’s recorded injuries, thereby improving precision in estimating overall injury severity. It is also more feasible for national-level research, as ICD coding is legally required in all medical records, unlike the Abbreviated Injury Scale (AIS), which lacks consistent nationwide implementation. Adaptation of ICISS to the ICD-10 system has further enhanced its accuracy^7,8^, and its predictive validity has been demonstrated in both European and Swedish trauma cohorts^3^.

Our research group has previously applied ICISS in national trauma analyses^3,4,9^. However, ICISS values derived from a pooled trauma cohort—combining cases of traffic, fall, and assault—may reduce predictive precision when applied to a particular subset, such as the traffic group. Therefore, the second aim of this study was to calculate ICISS specifically within the traffic-related trauma subgroup, representing the first such effort, to improve risk adjustment and refine outcome interpretation.

The impact of hospital level on survival after traffic-related trauma remains uncertain. In contrast to findings from North America^10–12^ and Finland^13^, our earlier Swedish study^4^ indicates that hospital type does not seem to influence risk-adjusted traffic-related mortality, using data from 2001 to 2011. It is now important to reassess this question using contemporary data, particularly in light of the recent emphasis on trauma system centralisation and reports suggesting a survival advantage at Level 1 hospitals^14^.

Traditionally, large registry analyses have relied on multivariable logistic regression (LR). However, artificial intelligence (AI) and, in particular, ensemble learning algorithms such as XGBoost have demonstrated superior performance in medical predictive modelling^15,16^. Consequently, the third and final aim of this study was to evaluate whether XGBoost can improve the performance of the ICISS-based mortality prediction model and enhance understanding of the underlying risk factors by utilising explainable AI techniques.

## Methods

### Patient cohort

The National Patient Register (NPR), which includes all hospital admissions in Sweden since 1987, was used to identify trauma-related admissions between 2008 and 2021. Each patient’s unique personal identification number (PIN) enabled linkage with the Cause of Death Register, ensuring complete follow-up for all recorded deaths among Swedish citizens.

Trauma cases were identified using the International Classification of Diseases, 10th Revision (ICD-10). Diagnoses within the S00–T79 range and external cause codes (E-codes, V01–Y98) were used to generate the initial trauma database. The code range T36–T79, excluding T689 (hypothermia), was omitted as it includes poisonings, intoxications, and other nonspecific adverse effects outside the trauma scope. To define the subgroup of traffic-related trauma, E-codes V01–V89 were used, excluding V80–V82 and V88.

The ICD-based Injury Severity Score (ICISS) was calculated both for the complete trauma dataset (including assaults and falls) and for the traffic-related subset, referred to as the *“Traffic database for calculation”*(see Figure A1, Appendix). All analyses in this study were based on the ICISS values derived from this traffic-related subset. Hospital admissions due to other trauma mechanisms or cases assigned to multiple trauma categories were excluded.

Finally, patients admitted to hospitals that did not meet the predefined hospital categorisation criteria (see below) were excluded. The resulting dataset comprised 95,954 hospital admissions for traffic-related trauma (Figure A1, Appendix).

### Hospital categorisation

The Swedish PeriOperative Registry (SPOR) classification of hospital level—recognised by the Swedish National Board of Health and Welfare (Socialstyrelsen), the Swedish Association of Local Authorities and Regions, the Swedish Association of Anaesthesia and Intensive Care, and the national healthcare insurance bodies—was used as the basis for hospital categorisation in this study due to its broad acceptance within the Swedish healthcare system^4,17^.

Hospitals were classified into three categories according to their level of intensive care capability, degree of specialised services, access to diagnostic resources (laboratory and radiology), and involvement in research and education:

- Level 1 hospitals: university hospitals,
- Level 2 hospitals: regional hospitals,
- Level 3 hospitals: county hospitals.

Detailed inclusion criteria are provided in Appendix Table A2. For Level 3 hospitals, the available SPOR classification could not be fully applied because several hospitals in this category were not included in the list. These hospitals were therefore identified and included through face validation, which involved direct communication with hospital leadership (medical directors or heads of operations), regional administrators, or comprehensive web-based verification. Hospitals for which emergency or trauma-related admissions during the study period could not be reliably confirmed were excluded from the analysis (Appendix Table A2).

### Injury severity

The traffic database (Figure A1, Appendix) was used to calculate the diagnosis-specific survival probability (DSP) for each ICD-10 code, representing the likelihood of survival associated with each specific injury. Duplicate ICD-10 codes were removed before DSP calculation. Using an inclusive approach, an ICISS value was then derived for each hospital admission as the product of the individual DSPs corresponding to that patient’s ICD-10 injury codes. Because the ICISS distribution was highly skewed, a logarithmic transformation was applied for regression analyses: *log10(1 − ICISS + ε)*, where *ε* = 0.001.

### Comorbidities

The Charlson Comorbidity Index (CCI), adapted for the ICD-10 system as described by Glasheen et al.^18^, was used in this study. The translation of ICD-9 codes followed the same approach outlined by Glasheen et al.^18^. The resulting mappings were cross-checked against the classifications of the Swedish National Board of Health and Welfare^19^ to ensure consistency with the ICD-9 coding system used in Sweden during the study period.

### Statistics

We applied both standard multivariable logistic regression and explainable artificial intelligence (XAI) methods, combining XGBoost and SHapley Additive exPlanations (SHAP), as implemented in the R packages *xgboost* and *SHAP*^20–22^. The dataset was randomly split into an 80% training set and a 20% test set, following widely accepted conventions in machine learning to ensure robust model development and unbiased performance evaluation. Model discrimination was assessed using the area under the receiver operating characteristic curve (AUC-ROC) on the test set.

For the XGBoost model, a grid search was performed on the training set to identify the optimal hyperparameters. The final configuration included: *objective* = “binary: logistic”, *learning_rate* = 0.1, *subsample* = 0.3, *colsample_bynode* = 0.3, *reg_lambda* = 6, *max_depth* = 50, *eval_metric* = “auc”, and *scale_pos_weight* = 99. ICISS scores were calculated separately for the training and test sets to prevent data leakage. The optimised XGBoost model was then trained on the training set and evaluated on the test set. Differences in AUC values between models were compared using the DeLong test^23^. Model interpretability was achieved through SHAP, which quantifies the contribution of each feature to individual predictions.

## Results

### Patient characteristics

**Fig. 1:**
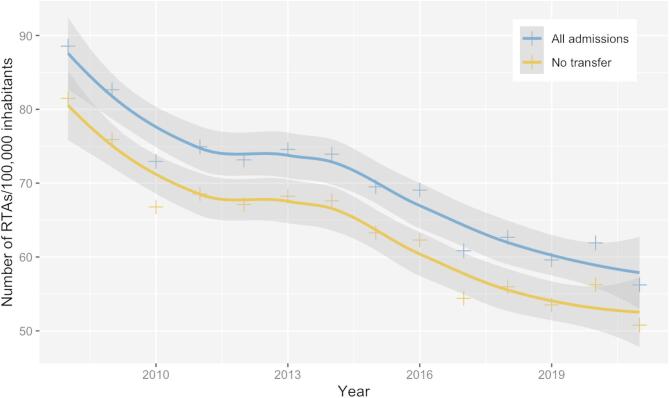
Annual traffic-related hospital admissions per 100,000 inhabitants. Annual number of hospital admissions related to road traffic accidents per 100,000 inhabitants^24^. The continuous lines represent local polynomial regression estimates, computed using the *loess* function in R (see *Statistics* section under *Methods*). The blue line shows all hospital admissions, while the yellow line represents admissions without inter-hospital transfer.

**Fig. 2:**
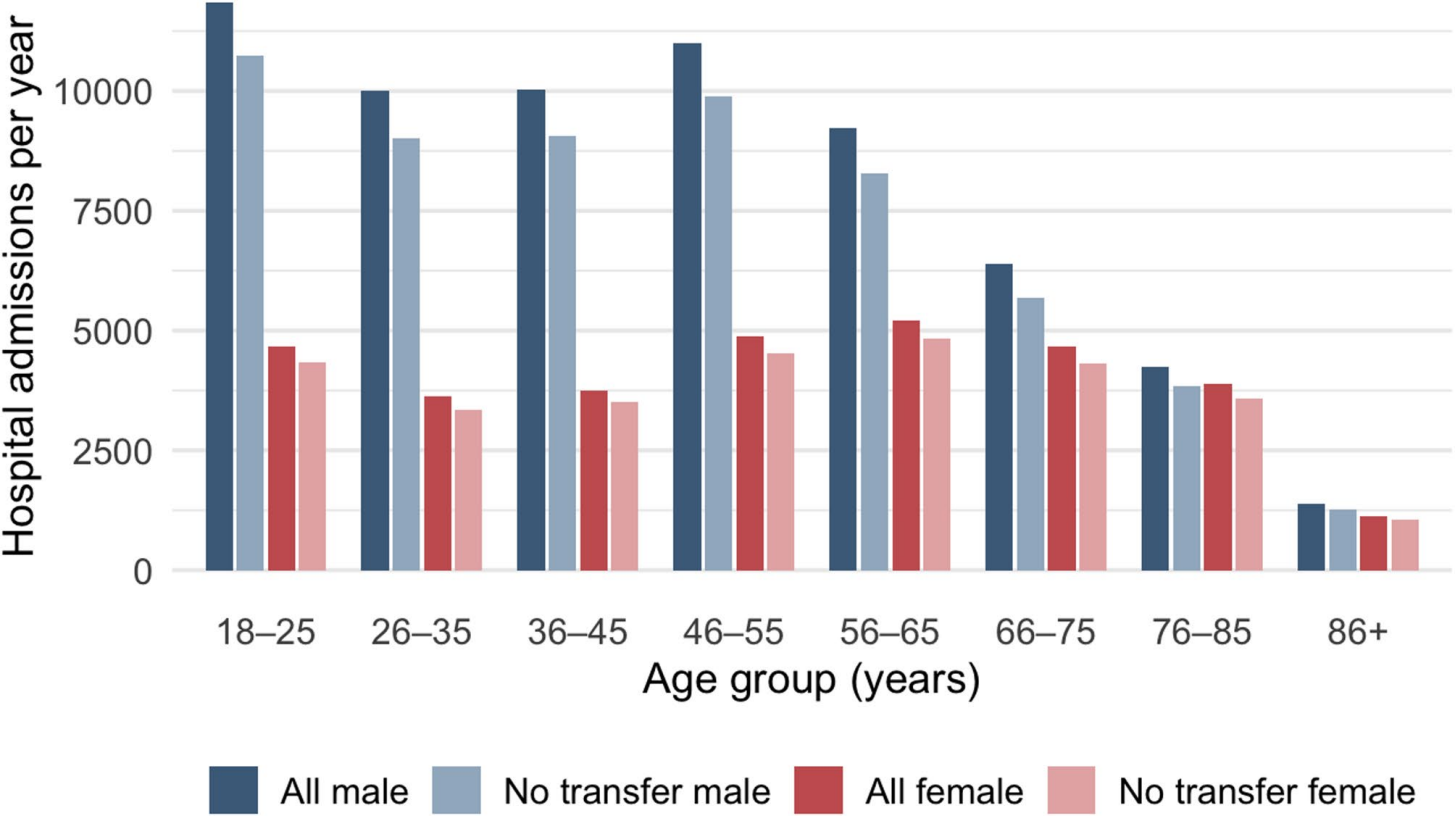
Road traffic accident hospital admissions by year, transfer status, and sex. Number of hospital admissions for road traffic accidents (RTAs) in Sweden from 2008 to 2021, stratified by sex and age group. Data are shown separately for all admissions and for cases without inter-hospital transfer.

The final study population comprised 95,954 hospital admissions between 2008 and 2021, with a 30-day mortality rate of 1.16% and a median ICISS of 0.91 (interquartile range [IQR] 0.11). The distribution of ICISS across hospital levels is presented in Table 1. Patient ages ranged from 18 to 103 years, with a median and mean of 48 years (IQR 33 years; SD 19 years). Men accounted for 67% of admissions and were overrepresented in all age groups (Table 1; Fig. 2). The annual incidence of hospitalised road-traffic accidents (RTAs) is shown in Fig. 1.

Among fatal cases, 46% were associated with traumatic brain injury (TBI), defined by ICD-10 codes S061–S069. In the elderly cohort (≥ 65 years), over 40% of deaths involved TBI. Temporal trends in patient characteristics are summarised in Table A1 (Appendix).

### Regression models

Figure A2 (Appendix) displays the receiver operating characteristic (ROC) curves and corresponding AUCs for the logistic regression (LR) and explainable AI (XAI) models predicting 30-day mortality (*p* < 0.05, DeLong’s test). Calibration, assessed using the Brier score, was 1.00 for the LR model and 0.09 for the XAI model (*p* < 0.05).

**Fig. 3:**
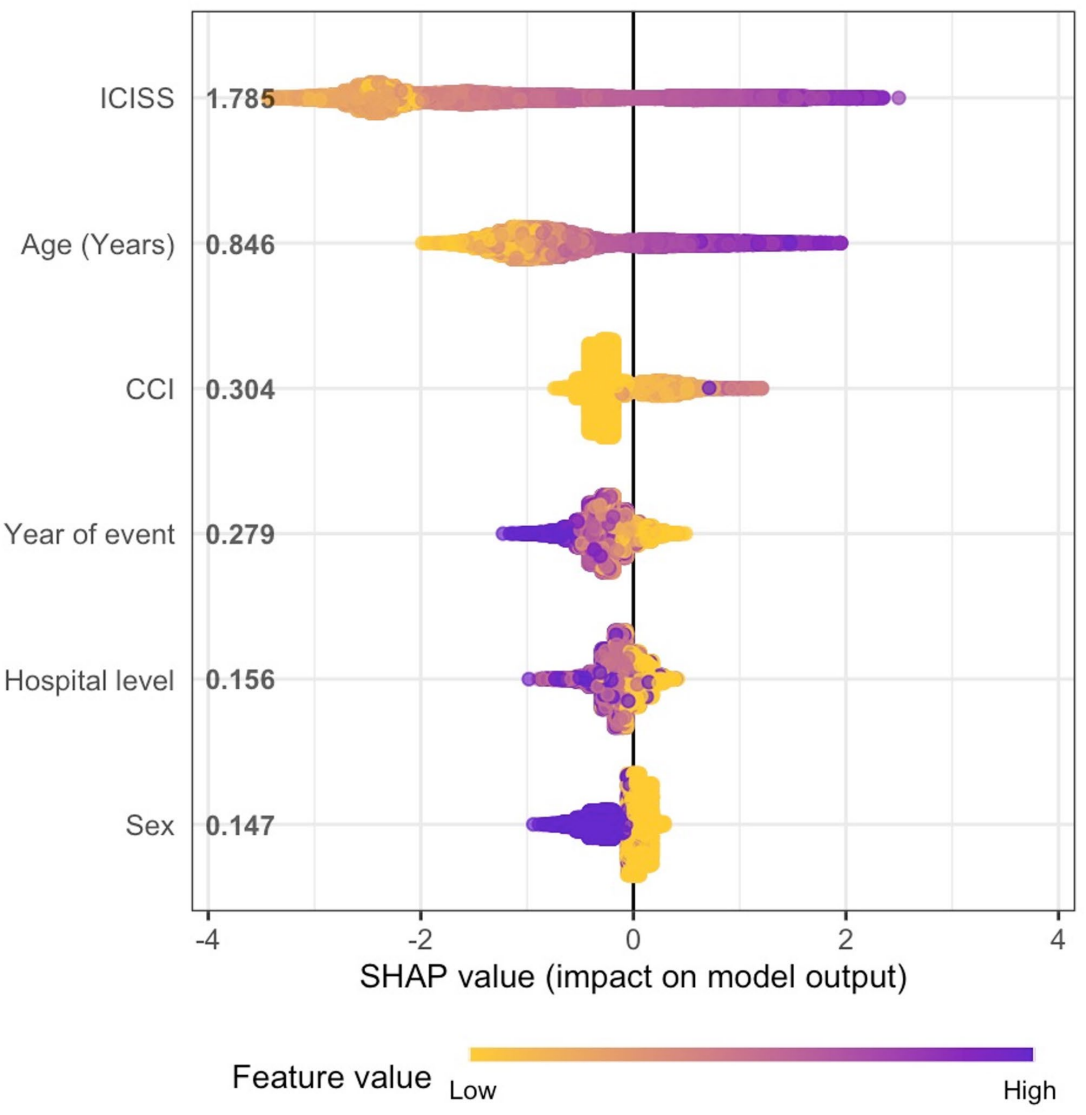
SHAP summary plot. Contribution of each variable to 30-day mortality prediction using mean absolute SHAP values. The x-axis represents the SHAP value (in log odds), with each dot corresponding to one patient. Colour intensity indicates the variable’s value, where darker tones denote higher values (e.g., age 80 is darker than age 50). The two most influential predictors were ICISS and age. Lower ICISS values (lighter tones toward the right) were associated with higher mortality risk, whereas younger age (lighter tones toward the left) indicated lower risk. For sex, male patients are represented in yellow. SHAP values for ICISS were derived from log₁₀(1 – ICISS + 0.001), resulting in an inverted colour scale.

Table 2 shows the odds ratios from the multivariable LR model including ICISS, age, sex, CCI, event year, and hospital level. In decreasing order of importance, the strongest predictors of 30-day mortality were ICISS, age, CCI, event year, hospital level, and sex, as visualised in the summary plot (Fig. 3).

**Fig. 4:**
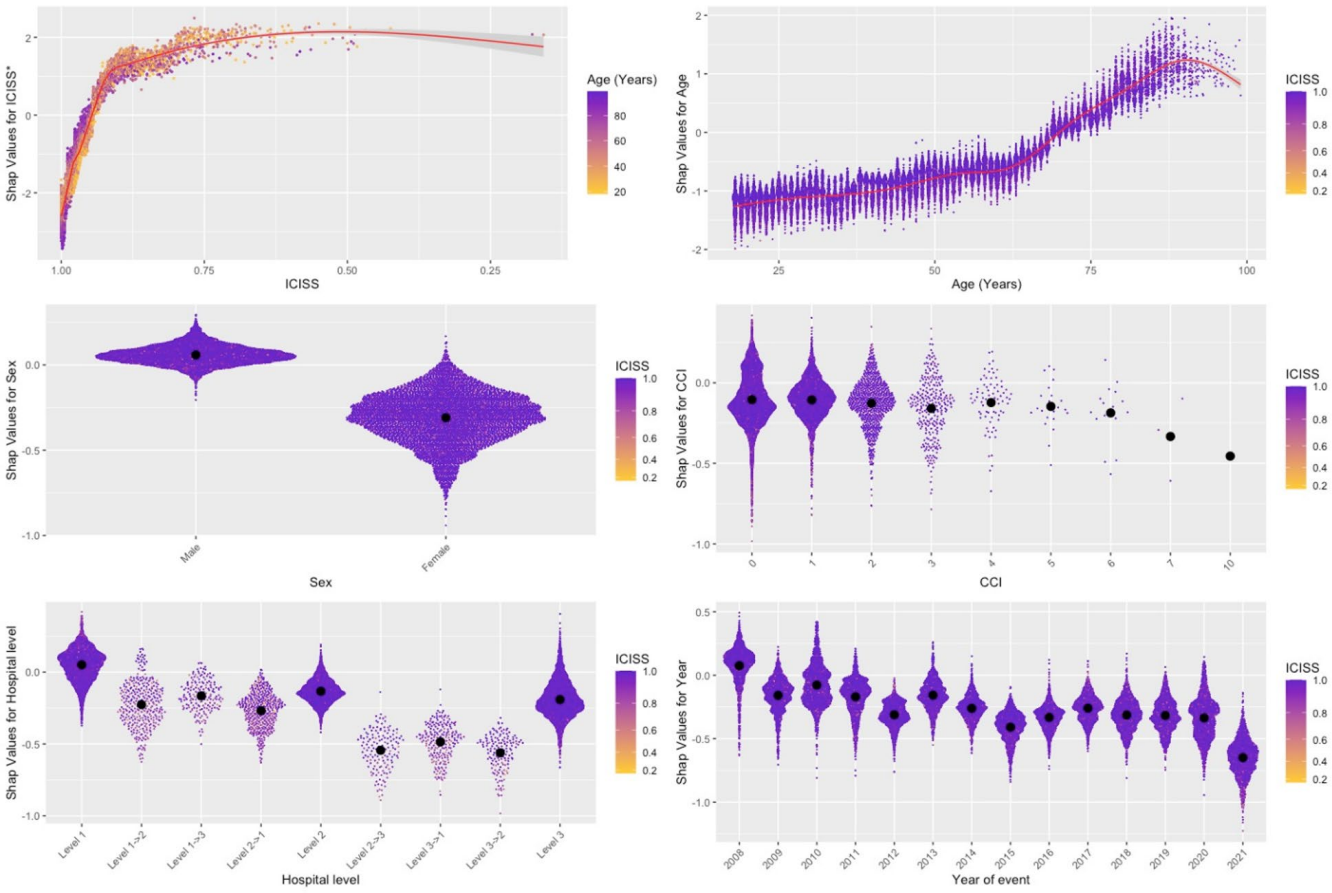
SHAP dependency plots. Top left (ICISS): Mortality risk increased sharply as ICISS decreased from 1.0 to 0.9, plateauing and peaking near 0.6. Top right (Age): Mortality risk rose almost linearly with age. Middle left (Sex): Male sex was associated with higher mortality than female sex. Middle right (Charlson Comorbidity Index, CCI): Minimal effect observed until CCI > 6. Bottom left (Hospital level): Among non-transferred patients, mortality risk ranked (highest to lowest): Level 1 > Level 3 > Level 2. Transferred patients exhibited a lower overall mortality risk. Bottom right (Year of event): Risk decreased in 2009 compared with 2008, stabilised thereafter, and declined again from 2018 to 2021. *ICISS* ICD-10 Injury Severity Score, *CCI* Charlson Comorbidity Index. SHAP values for ICISS were calculated from log₁₀(1 – ICISS + 0.001).

**Fig. 5:**
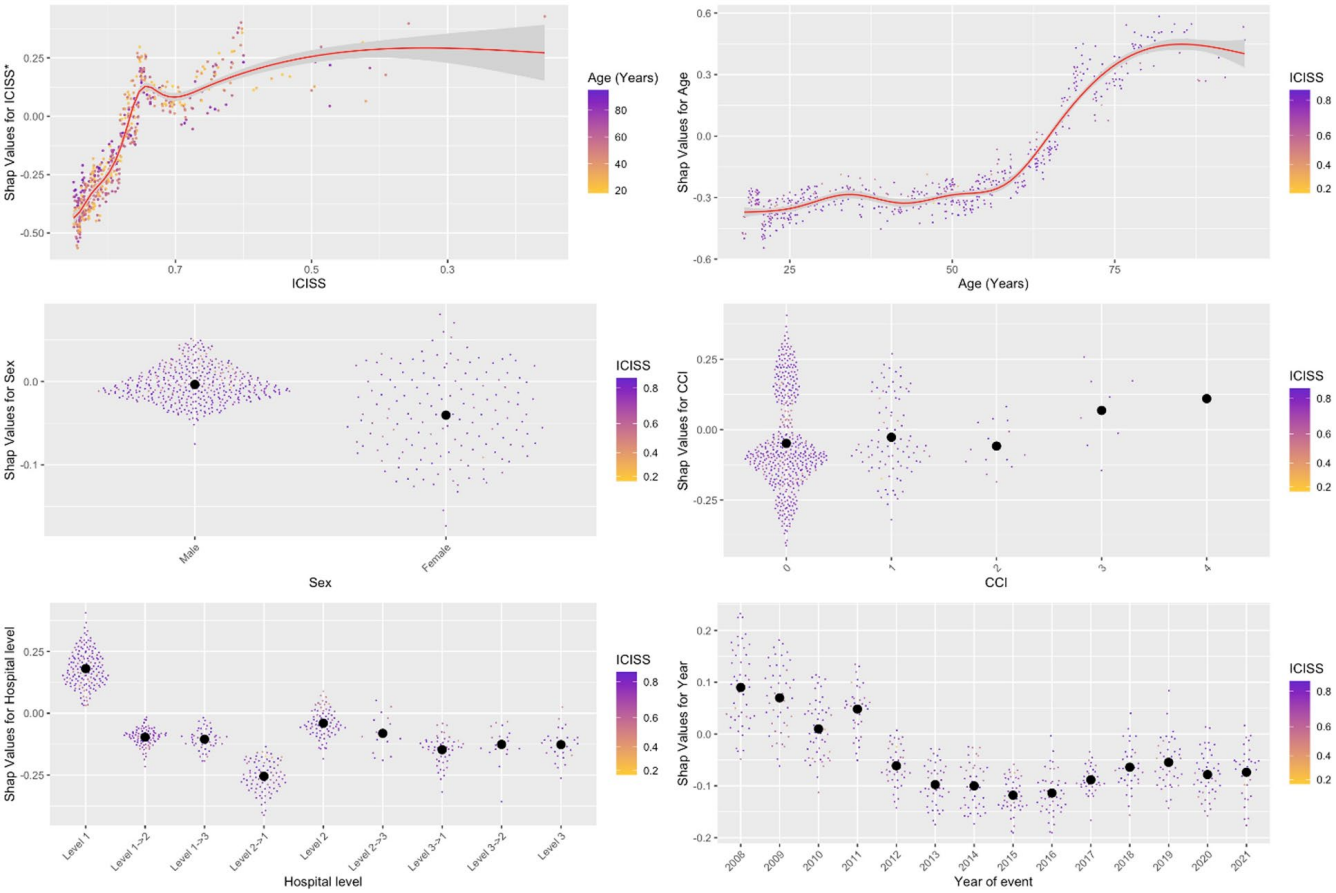
SHAP dependency plots for severely injured patients (ICISS ≤ 0.85). Top left (ICISS): Mortality increased with decreasing ICISS, peaking around 0.57. Top right (Age): Mortality risk began to rise from approximately 50 years of age. Middle left (Sex): Male patients had a higher mortality risk than female patients. Middle right (CCI): Mortality increased progressively with higher comorbidity scores. Bottom left (Hospital level): Transferred patients had lower mortality overall. Among non-transferred patients, Level 1 hospitals were associated with the highest mortality, followed by Levels 2 and 3. Bottom right (Year of event): Mortality risk decreased steadily over time. ICISS = ICD-10 Injury Severity Score; CCI = Charlson Comorbidity Index. SHAP values for ICISS were calculated from log₁₀(1 – ICISS + 0.001).

Lower ICISS values were independently associated with higher mortality (Table 2; Figs. 4 and 5). Although ICISS declined slightly over time, the magnitude of this change was small (Table A1). Increasing age was independently associated with higher mortality (Table 2; Figs. 4 and 5), and the median age of patients increased notably over the study period (Table A1). Similarly, a higher CCI was associated with increased mortality, with substantial variation in CCI observed over time (Table A1).

Thirty-day mortality was highest at Level 1 hospitals, followed by Levels 2 and 3. For the most severely injured and non-transfer patients, the hospital level was not an independent predictor of mortality. Subgroup analysis restricted to transfer patients (data not shown) revealed no significant differences between hospital levels in the LR model. Consistently, the XAI model indicated higher mortality at Level 1 hospitals and similar outcomes at Levels 2 and 3, both for the overall and severely injured cohorts (Figs. 4 and 5).

Use of Level 2 hospitals increased over time, corresponding to a decrease in Level 3 admissions (Table A1).

Female sex was independently associated with lower 30-day mortality across all admissions and among non-transfer patients (Table 2; Figs. 4 and 5).

Finally, the adjusted 30-day mortality rate decreased over the study period for both the total cohort and the non-transfer subgroup (Table 2; Figs. 4 and 5).

## Discussion

By applying explainable artificial intelligence (XAI) to road traffic accident (RTA) data combined with ICD-10–based injury severity scoring (ICISS), we demonstrated that the XAI model outperformed conventional multivariable logistic regression in predicting 30-day mortality following RTA hospitalisation. The enhanced performance of the XAI model enabled a more detailed and interpretable characterisation of risk factors associated with RTA-related 30-day mortality.

### Injury severity

In the XAI model, the most influential predictors of 30-day mortality, in descending order of importance, were ICISS, age, CCI, event year, hospital level, and sex. As expected, injury severity, as represented by ICISS, was the primary determinant of mortality. The ICISS values in this study were higher than those reported by Osler et al.^6^ (median 0.93) and Larsen et al.^9^ (median 0.95). This difference likely reflects that our estimates are based solely on RTA cases, rather than on all trauma admissions. The comparatively higher ICISS values are further illustrated by the median ICISS of 0.91 among fatal cases, compared with 0.45 in Osler’s study^6^ and 0.72 in Ydenius et al.^4^. The ageing demographic profile of RTA patients^25^ is also a likely contributing factor^26^. In Osler’s and Ydenius’s cohorts, 92% and 77% of patients, respectively, were 55 years of age or younger, whereas our dataset includes a substantially older population.

In our study, the median ICISS was 0.877 among individuals aged 18–25 years and 0.935 among those aged 75 years or older (data not shown). Table 1 demonstrates that severe cases, defined as ICISS < 0.85, were uncommon, comprising approximately 3% of hospital admissions. This raises the question of whether the conventional ICISS cutoff of 0.85 for severe RTA may be overly restrictive, as many fatalities in this dataset occurred at higher ICISS levels. Notably, a threshold of < 0.941 has previously been suggested to define severe injury^27^.

### Road traffic accident victims over time

The temporal analysis of patient demographics (Table A1, Appendix) shows that the median age of RTA victims increased over time, while the median ICISS decreased, indicating a trend toward more severe injuries. The Charlson Comorbidity Index (CCI) also varied significantly across the study period. Moreover, a smaller proportion of patients were treated at Level 3 hospitals, with a corresponding shift toward Level 2 facilities. Collectively, these factors would be expected to contribute to an overall increase in mortality risk.

However, as shown in Table 2 and in the lower-right panels of Figs. 4 and 5, the adjusted mortality risk actually declined over time, suggesting improvements in trauma care or system-level efficiency despite the increasing severity of injuries and the growing comorbidity burden.

### Age

Both younger and older individuals are disproportionately affected by fatal road traffic accidents (RTAs)^28^. Among younger people, the increased risk is often attributed to risk-prone behaviour^29,30^. This pattern is reflected in our cohort, where the median age of RTA-related hospital admissions was 47 years (IQR 32), and young men were clearly overrepresented (Fig. 2). In contrast, the median age among fatalities was substantially higher at 72 years (IQR 31), consistent with our findings that mortality increases with advancing age (Table 2; Fig. 4). It has been suggested that younger individuals involved in fatal RTAs are more likely to die at the scene or during transport, thereby not being captured in in-hospital mortality analyses.

### Sex and comorbidity

Previous studies have shown that female patients tend to have a survival advantage compared with males^31^ and that comorbidity is an important determinant of 30-day mortality^4,9^. In our logistic regression model restricted to the most severely injured patients, the effects of sex and comorbidity were small and not statistically significant, likely reflecting limited statistical power due to the small number of observations. In contrast, the XAI model revealed a subtle sex-related difference and an increased mortality risk for patients with a Charlson Comorbidity Index (CCI) of 1 compared with those with a CCI of 0 among the severely injured subgroup.

Notably, very few individuals with a CCI greater than 6 (on a scale from 0 to 20) were admitted to hospital with RTA-related trauma (Fig. 4). This observation may indicate that individuals with extensive comorbidity are less frequently exposed to traffic situations that lead to severe injury^32^.

### Hospital level

The most severely injured patients are predominantly admitted to Level 1 hospitals, where crude mortality is higher than at Levels 2 and 3 (Tables 1 and 2; Figs. 4 and 5), consistent with the findings of Ydenius et al.^4^. However, in the logistic regression model, higher hospital level (Level 1 > Level 2 > Level 3) was not independently associated with improved 30-day mortality among either the most severely injured or non-transfer patients. In contrast, the XAI model suggested an apparent survival advantage for lower hospital levels. As formal statistical testing for this observation cannot be performed, we refrain from concluding that patients treated at lower-level hospitals experience better adjusted outcomes. Earlier criticisms about attributing outcomes to the admitting hospital have been addressed by categorising patients based on transfer status.

The long-held belief that early trauma care—the so-called “Golden Hour”—is the principal determinant of trauma outcomes has increasingly been questioned^33^. Recent North American studies instead emphasise the benefits of structured trauma centre referral systems rather than focusing solely on the timing of initial care^34,35^. Our findings, showing no survival advantage for Level 1 centres, raise the broader question of whether Swedish trauma care should prioritise centralised referral pathways or rapid access to the nearest hospital. Candefjord et al.^14^ argue that, in theory, most trauma patients in Sweden could reach a trauma centre within one hour if helicopter transport were widely available. In practice, however, helicopter ambulance resources remain limited. An alternative approach may therefore lie in “bringing the emergency room to the patient” through advanced, well-equipped ground ambulances^36^.

Transfers between hospitals further complicate the interpretation of hospital-level effects. In this study, patients were categorised as either non-transferred or transferred (based on the first transfer event). Notably, the vast majority of admissions (91%) were not transferred to another facility. Because early deaths inherently preclude transfer, this introduces selection bias that must be acknowledged. Future work should include formal statistical analysis of SHAP values to quantify the relative importance of hospital-level and related features in determining outcomes.

### Improving methods

Previous research has shown that gradient boosting algorithms, such as XGBoost, can enhance trauma mortality prediction compared to traditional logistic regression models based on the Injury Severity Score (ISS), the Trauma Mortality Prediction Model (TMPM-ICD10), and the Trauma and Injury Severity Score (TRISS)^37,38^. In the present study, we applied a novel approach by calculating the ICISS specifically for road traffic accident (RTA) cases and integrating it into a machine learning framework. This method improved risk adjustment and mortality prediction, with the AUC increasing from 0.90 using logistic regression to 0.92 with the explainable AI (XAI) model. To our knowledge, the application of XAI to ICISS data is original and provides deeper insight into the complex, non-linear relationships underlying trauma outcomes.

### Limitations

Although the SweTrau registry was initially considered as a potential data source, it was established after the onset of this study. Consequently, the National Patient Register (NPR) was selected. Legal requirements for ICD coding substantially reduce the risk of missing data. While incomplete or inaccurate coding can never be entirely excluded, financial incentives for accurate coding and previous validation support good precision for ICD codes up to the fourth character^3,9^. Accordingly, the International Classification of Diseases Injury Severity Score (ICISS) represents a robust approach for risk adjustment in large administrative datasets^7^ and is generally preferred over the traditional ISS method^39^. Ydenius et al. have previously demonstrated the validity of the ICISS approach, and our findings further confirm its utility in a contemporary national context. The dataset’s size, inclusion period, and population-based nature—reflecting real-world conditions—constitute important strengths.

Patients who died at the scene or during transport to the hospital were not included in this study. Data from Transport Analysis (TRAFA)^40^ indicate higher survival rates in the metropolitan regions of Stockholm, Gothenburg, and Malmö. At the same time, mortality remains higher in sparsely populated northern areas—suggesting that transport time may influence pre-hospital outcomes. However, as this study focuses on *in-hospital* admissions, transport time lies outside its analytical scope and would require a separate investigation.

Using an anatomical risk-adjustment tool such as ICISS means that dynamic physiological factors influencing outcome may be underrepresented^41–43^. Physiological variables on arrival to the emergency department are, in turn, shaped by pre-hospital stabilisation. Nevertheless, the derived survival probability (DSP) calculated retrospectively still reflects these physiological effects, as severe anatomical injury typically coincides with deranged vital signs.

The analysis of hospital-level effects is further complicated by selection bias, since the most severely injured patients—those who die early—are unlikely to be eligible for transfer to higher-level hospitals. The transferred group, comprising roughly 9% of the dataset, is heterogeneous, as transfer timing is not specified and may occur immediately after trauma or weeks later. Caution is therefore warranted when interpreting results from this subgroup. However, distinguishing between transferred and non-transferred patients also allowed us to isolate the hospital level as an independent risk factor for 30-day mortality among non-transferred cases.

Explainable AI (XAI) techniques add complementary value by visualising feature importance and complex interactions that traditional models may overlook. While XAI methods such as SHAP currently lack formal inferential testing, combining them with conventional logistic regression provides both interpretability and statistical rigour. Ongoing research aims to develop computational methods for statistical inference with XGBoost and SHAP, though this lies beyond the scope of the present study.

## Conclusion

An explainable AI (XAI) model for predicting 30-day mortality among hospitalised road traffic accident (RTA) patients—based on ICD-coded injury severity (ICISS), age, Charlson Comorbidity Index (CCI), event year, hospital level, and sex (in decreasing order of importance)—demonstrated superior discrimination and calibration compared with a corresponding logistic regression (LR) model. Despite increasing injury severity and a rise in median patient age from 44 to 52 years between 2008 and 2021, adjusted mortality declined over the study period, suggesting improvements in trauma care and treatment efficacy.

Sex and comorbidity were associated with 30-day mortality but did not reach statistical significance among the most severely injured patients (ICISS ≤ 0.85). The ongoing trend toward centralisation of trauma care at Level 1 hospitals persisted, accompanied by a decreasing gradient of injury severity from Level 1 to Level 3 hospitals. However, after adjustment for relevant covariates, hospital level was not independently associated with mortality, particularly among the most severely injured.

A major strength of this study lies in the stratification by transfer status and the robust use of ICISS as a validated, objective measure of injury severity. Future studies assessing hospital-level effects should incorporate transport time and pre-hospital factors to more accurately reflect the full continuum of trauma care. Additionally, expanding the XAI framework to include formal statistical testing would enhance interpretability and generalisability.

In conclusion, this study challenges prevailing assumptions regarding the benefits of trauma system centralisation, indicating that, within the Swedish healthcare context, higher hospital level does not confer a survival advantage for RTA patients.

The datasets analysed in the current study are available from the corresponding author upon reasonable request.

**Table 1:** Patient characteristics stratified by injury severity, transfer status, and hospital level.

Variable	All Patients					Severely Injured (ICISS ≤ 0.85)			No transfer				Severely Injured, No transfer			
	Level 1	Level 2	Level 3	Total	Level 1	Level 2	Level 3	Total	Level 1	Level 2	Level 3	Total	Level 1	Level 2	Level 3	Total
Cases (***n*)**	31 431	42 977	21 546	**95 954**	1 380	1 000	439	**2 819**	28 186	39 563	19 525	**87 274**	571	271	89	**931**
	*33%*	*45%*	*23%*	***100%***	*49%*	*35%*	*16%*	***100%***	*32%*	*45%*	*22%*	***100%***	*61%*	*29%*	*10%*	***100%***
Male	21 180	28 550	14 405	**64 135**	1 009	730	317	**2 056**	18 803	26 051	12 945	**57 799**	402	173	66	**641**
	*67%*	*66%*	*67%*	***67%***	*73%*	*73%*	*72%*	***73%***	*67%*	*66%*	*66%*	***66%***	*70%*	*64%*	*74%*	***69%***
Female	10 251	14 427	7141	**31 819**	371	270	122	**763**	9 383	13 512	6580	**29 475**	169	98	23	**290**
	*33%*	*34%*	*33%*	***33%***	*27%*	*27%*	*28%*	***27%***	*33%*	*34%*	*34%*	***34%***	*30%*	*36%*	*26%*	***31%***
Age (years), median	47	49	49	**48**	48	49	51	**48**	47	49	49	**48**	50	56	55	**52**
	*IQR 32*	*IQR 33*	*IQR 36*	***IQR 33***	*IQR 34*	*IQR 35*	*IQR 32*	***IQR 34***	*IQR 32*	*IQR 33*	*IQR 36*	***IQR 33***	*IQR 30*	*IQR 33*	*IQR 33*	***IQR 36***
ICISS, median	0.988	0.991	0.991	**0.990**	0.785	0.782	0.778	**0.784**	0.990	0.992	0.993	**0.992**	0.788	0.796	0.784	**0.792**
	*IQR 0.036*	*IQR 0.023*	*IQR 0.023*	***IQR 0.025***	*IQR 0.093*	*IQR 0.094*	*IQR 0.086*	***IQR 0.092***	*IQR 0.026*	*IQR 0.019*	*IQR 0.017*	***IQR 0.021***	*IQR 0.091*	*IQR 0.074*	*IQR 0.052*	***IQR 0.080***
Age (years)																
18–25	5315	7 172	4 046	**16 533**	281	172	74	**527**	4753	6 591	3 731	**15 075**	100	40	13	**153**
	32%	43%	24%	**17%**	53%	33%	14%	**19%**	32%	44%	25%	**17%**	65%	26%	8%	**16%**
26–35	4857	5988	2770	**13 615**	199	134	43	**376**	4378	5492	2507	**12 377**	81	26	8	**115**
	*36%*	*44%*	*20%*	***14%***	*53%*	*36%*	*11%*	***13%***	*35%*	*44%*	*20%*	***14%***	*70%*	*23%*	*7%*	***12%***
36–45	4732	6145	2917	**13 794**	165	132	57	**354**	4255	5676	2628	**12 559**	66	26	9	**101**
	*34%*	*45%*	*21%*	***14%***	*47%*	*37%*	*16%*	***13%***	*34%*	*45%*	*21%*	***14%***	*65%*	*26%*	*9%*	***11%***
46–55	5465	7120	3288	**15 873**	250	167	78	**495**	4903	6552	2963	**14 418**	96	39	16	**151**
	*34%*	*45%*	*21%*	***17%***	*51%*	*34%*	*16%*	***17%***	*34%*	*45%*	*21%*	***17%***	*64%*	*26%*	*11%*	***16%***
56–65	4816	6525	3082	**14 423**	219	156	76	**451**	4319	6010	2779	**13 108**	95	50	13	**158**
	*33%*	*45%*	*21%*	***15%***	*49%*	*35%*	*17%*	***16%***	*33%*	*46%*	*21%*	***15%***	*60%*	*32%*	*8%*	***17%***
66–75	3269	5067	2728	**11 064**	150	124	61	**335**	2880	4651	2453	**9 984**	60	39	13	**112**
	*30%*	*46%*	*25%*	***12%***	*45%*	*37%*	*18%*	***12%***	*29%*	*47%*	*25%*	***11%***	*54%*	*35%*	*12%*	***12%***
76–85	2246	3800	2089	**8 135**	91	92	36	**219**	2025	3497	1901	**7 423**	59	39	12	**110**
	*28%*	*47%*	*26%*	***8%***	*42%*	*42%*	*16%*	***8%***	*27%*	*47%*	*26%*	***9%***	*54%*	*35%*	*11%*	***12%***
≥ 86	731	1160	626	**2 517**	25	23	14	**62**	673	1094	563	**2 330**	14	12	5	**31**
	*29%*	*46%*	*25%*	***3%***	*40%*	*37%*	*23%*	***2%***	*29%*	*47%*	*24%*	***3%***	*45%*	*39%*	*16%*	***3%***

Data are presented as absolute numbers and percentages, with percentages rounded to the nearest whole number. Demographic characteristics are shown for: (i) all hospital admissions; (ii) severely injured cases (as defined by ICISS); and (iii) non-transferred cases. The row labelled *Cases* indicates the distribution of patients across hospital levels. Percentages in the *Male* and *Female* rows represent the sex distribution within each hospital level and sum to 100% in the *Total* column. For age group categories, percentages represent the distribution across hospital levels. The *Total* column denotes the overall number of patients in each subgroup: 95,954 for all cases, 2,819 for severely injured cases, and 87,274 for non-transferred cases.

*ICISS* ICD-10 Injury Severity Score, *IQR* Interquartile Range.

**Table 2:** Multivariable logistic regression for 30-day in-hospital mortality.

Variable	OR	*p*-value	90% CI
All Admissions			
ICISS*	15.24	< 0.001	13.28–17.49
Age (Years)	1.04	< 0.001	1.04–1.05
Sex (Female)	0.77	< 0.001	0.67–0.89
CCI	1.34	< 0.001	1.26–1.42
Year	0.96	< 0.001	0.95–0.98
Hospital Level 2	0.84	0.012	0.73–0.96
Hospital Level 3	0.77	0.004	0.65–0.92
Severely Injured (ICISS ≤ 0.85)			
ICISS*	53.49	< 0.001	21.24–136.66
Age (Years)	1.03	< 0.001	1.03–1.04
Sex (Female)	0.92	0.60	0.69–1.24
CCI	1.18	0.09	0.97–1.42
Year	0.97	0.05	0.94–1.00
Hospital Level 2	0.81	0.16	0.61–1.08
Hospital Level 3	0.67	0.05	0.46–1.00
No Transfer			
ICISS*	22.32	< 0.001	18.90–26.36
Age (Years)	1.04	< 0.001	1.04–1.04
Sex (Female)	0.79	0.003	0.68–0.92
CCI	1.3	< 0.001	1.21–1.38
Year	0.96	< 0.001	0.94–0.98
Hospital Level 2	0.88	0.09	0.75–1.02
Hospital Level 3	0.86	0.13	0.71–1.04
No Transfer & Severely Injured (ICISS ≤ 0.85)			
ICISS*	270.21	< 0.001	60.93–1198.25
Age (Years)	1.02	< 0.001	1.01–1.03
Sex (Female)	0.84	0.39	0.56–1.25
CCI	0.99	0.94	0.75–1.30
Year	0.97	0.26	0.93–1.02
Hospital Level 2	1.09	0.68	0.73–1.64
Hospital Level 3	0.91	0.76	0.48–1.71

Estimates are presented as odds ratios (OR) with 95% confidence intervals (CI). The variable *log₁₀(1 – ICISS + 0.001)*was used in the model to improve interpretability and model fit.

*ICISS* ICD-10 Injury Severity Score, *CCI* Charlson Comorbidity Index, *CI* confidence interval, *OR* odds ratio.

## Supplementary Information

Below is the link to the electronic supplementary material.

Supplementary Material 1

Supplementary Material 2

## Data Availability

The datasets analysed in the current study are available from the corresponding author upon reasonable request.

## Abbreviations

AI: Artificial Intelligence
AIS: Abbreviated Injury Scale
AUC: Area Under the Curve
CCI: Charlson Comorbidity Index
CI: Confidence Interval
DSP: Diagnosis-Specific Survival Probability
E-codes: External Cause of Injury Codes
ICD: International Classification of Diseases
ICD-10: International Classification of Diseases version 10
ICISS: ICD-based injury severity score
IQR: Interquartile Range
ISS: Injury Severity Score
LR: Multivariable Logistic Regression
NPR: National Patient Registry
OR: Odds Ratio
PIN: Personal Identification Number
ROC: Receiver Operating Characteristic curve
RTA: Road Traffic Accident
SHAP: SHapley Additive exPlanations
SPOR: Swedish PeriOperative Registry
TBI: Traumatic Brain Injury
TMPM-ICD10: Trauma Mortality Prediction Model based on International Classification of Diseases version 10
TRAFA: Transport Analysis
TRISS: Trauma and Injury Severity Score
XAI: Explainable Artificial Intelligence
XGBoost: Extreme Gradient Boosting
