# Supplementary material for: Higher hospital level does not improve 30-day survival after road traffic accidents
Source: Sci Rep. 2025 Nov 21;15:41164. doi: 10.1038/s41598-025-26519-7 (PMC12638988; doi:10.1038/s41598-025-26519-7)
Supplement: Supplementary file 1 — Supplementary Material 1 [file 41598_2025_26519_MOESM1_ESM.docx]

| **Hospital categorization by SPOR** | | |
| --- | --- | --- |
| **Category** | **Hospital Level** | **Definition** |
| **1** | **Level 1** | Access to all major medical specialities is available, including neurosurgery, thoracic surgery, paediatric anaesthesia, and infectious disease services. Advanced imaging and laboratory services are provided around the clock, alongside comprehensive intensive care. While certain sub-specialty units may be absent, the facility is classified as Intensive Care Category III. The surgical department may be geographically separate from the main university hospital but operates under the same administrative structure. The hospital also maintains a strong commitment to research and education as part of its core mission. |
| **2** | **Level 2** | Access is provided to all major medical specialities except neurosurgery, thoracic surgery, and paediatric anaesthesia. An infectious diseases clinic is available. Advanced imaging and laboratory services are offered around the clock. The facility is classified as Intensive Care Category II, although some sub-specialty units may be absent. |
| **3** | **Level 3** | Access to multiple medical specialities is available 24 hours a day, supported by X-ray and laboratory services for most of the day. An Intensive Care Category I unit may be present, though it is not a requirement. Maternal care services are provided. |
| **4** | **Level 3 without maternal care** | Limited access to medical specialities is available throughout the day, every day. X-ray and laboratory services are accessible for most of the day. An Intensive Care Category I unit may be present, but is not required. Maternal care services are not provided. |
| **5** | **Smaller Hospital Unit** | Access is limited to one or a few medical specialities, with no or only partial availability during night-time hours. |
| **8** | **Surgical activity data unavailable** | Surgical activity data unavailable |
| **9** | **No Surgery** | No surgical procedures are performed at this facility, irrespective of its size. |

**Intensive Care Category III**
Units in this category provide the highest level of supervision and treatment for various forms of organ failure. They fulfil all organisational, competence, and resource requirements for Category III. In addition to general intensive care, these departments often include highly specialised units—for example, those dedicated to thoracic surgery, neurosurgery, paediatrics, or severe burns. Hospitals with Category I and II units refer patients to Category III centres to ensure access to the most advanced level of care. Specialised intensive care units are typically geographically separate from general units and must independently meet all Category III criteria; otherwise, they are reclassified to a lower category.

**Intensive Care Category II**
Units in this category provide comprehensive care for most types of organ failure, particularly circulatory and respiratory failure. However, they do not offer the full range of supervision and treatment available in Category III. These units meet all organisational, competence, and resource requirements for Category II. They maintain a robust and safe referral system to Category III intensive care when escalation of care is required.

**Intensive Care Category I**
Units in this category provide appropriate care for selected types of organ failure but do not meet the criteria for Category II or III. They satisfy all organisational, competence, and resource requirements for Category I. A structured referral pathway to Category II or III units is in place to ensure timely access to higher levels of care when needed.
