# Supplementary material for: Higher hospital level does not improve 30-day survival after road traffic accidents
Source: Sci Rep. 2025 Nov 21;15:41164. doi: 10.1038/s41598-025-26519-7 (PMC12638988; doi:10.1038/s41598-025-26519-7)
Supplement: Supplementary file 2 — Supplementary Material 2 [file 41598_2025_26519_MOESM2_ESM.docx]

| Year | 2008-2010 | 2011-2014 | 2015-2018 | 2019-2021 | *p-value* |
| --- | --- | --- | --- | --- | --- |
| Patients/Year | 7589 | 7117 | 6568 | 6149 |  |
| Age (Median) (25-75 percentile) | 44 (28-61) years | 47 (29-63) years | 50 (32-65) years | 52 (34-67) years | <0.001† |
| Male (%) | 15095 (66) | 19020 (67) | 17574 (67) | 12446 (67) | 0.100†† |
| ICISS (Median) (25-75 percentile) | 0,9921 (0.9757-0.9967) | 0,9915 (0.9732-0.9967) | 0,9887 (0.9686 -0.9962) | 0,9887 (0.9639-0.9961) | <0.001† |
| CCI (mean) (SD) | 0,312 (0.738) | 0,293 (0.672) | 0,319 (0.707) | 0,305 (0.689) | <0.001†† |
| Level 1 (%) | 7601 (33) | 9337 (33) | 8411 (32) | 6082 (33) | <0.001†† |
| Level 2 (%) | 9676 (43) | 12358 (43) | 12092 (46) | 8851 (48) |  |
| Level 3 (%) | 5489 (24) | 6774 (24) | 5768 (22) | 3515 (19) |  |
| Level 1 (No transfer) (%) | 6881 (33) | 8419 (32) | 7491 (32) | 5395 (32) | <0.001†† |
| Level 2 (No transfer) (%) | 8980 (43) | 11468 (44) | 10983 (46) | 8132 (49) |  |
| Level 3 (No transfer) (%) | 5042 (24) | 6170 (24) | 5176 (22) | 3137 (19) |  |

**Table A1. Patient demographics over time.** Continuous variables were compared using the Kruskal–Wallis test (†), and categorical variables using the χ² test (††). **Abbreviations:**ICISS – ICD-10 Injury Severity Score; CCI – Charlson Comorbidity Index; SD – Standard Deviation.

| **Hospital level** |  | **Name of Hospital** |  | **Info** |
| --- | --- | --- | --- | --- |
| Level 1 Hospitals |  |  |  |  |
|  |  | Akademiska Sjukhuset^1^ |  |  |
|  |  | KS Huddinge^1^ |  |  |
|  |  | KS Solna^1^ |  |  |
|  |  | NKS Solna^1^ |  | ***i*** |
|  |  | Norrlands universitetssjukhus^1^ |  |  |
|  |  | Sahlgrenska^1^ |  | ***i*** |
|  |  | SUS Lund^1^ |  |  |
|  |  | SUS Malmö^1^ |  |  |
|  |  | Universitetssjukhuset i Linköping^1^ |  |  |
|  |  | Universitetssjukhuset Örebro^1^ |  |  |
|  |  |  |  |  |
| Level 2 Hospitals |  |  |  |  |
|  |  | Blekingesjukhuset^1^ |  | ***i*** |
|  |  | Capio S:t Görans Sjukhus AB^1^↑ |  |  |
|  |  | Centrallasarettet Växjö^1^ |  |  |
|  |  | Centralsjukhuset Karlstad^1^ |  |  |
|  |  | Centralsjukhuset Kristianstad^1^ |  |  |
|  |  | Danderyds Sjukhus^1^↓ |  |  |
|  |  | Falu lasarett^1^ |  |  |
|  |  | Gävle Sjukhus^1^ |  |  |
|  |  | Hallands Sjukhus Halmstad^1^ |  | ***i*** |
|  |  | Helsingborgs lasarett^1^ |  |  |
|  |  | Länssjukhuset i Kalmar^1^ |  |  |
|  |  | Länssjukhuset Ryhov^1^ |  |  |
|  |  | Mälarsjukhuset^1^ |  |  |
|  |  | Skaraborgs sjukhus^1^ |  | ***i*** |
|  |  | Sunderby Sjukhus^1^ |  |  |
|  |  | Sundsvalls sjukhus^1^ |  |  |
|  |  | SÄS Borås^1^ |  |  |
|  |  | Södersjukhuset^1^ |  |  |
|  |  | Vrinnevisjukhuset i Norrköping^1^ |  |  |
|  |  | Västmanlands Sjukhus Västerås^1^ |  |  |
|  |  | Östersunds Sjukhus^1^ |  |  |
|  |  |  |  |  |
|  |  |  |  |  |
| Level 3 Hospitals |  | Arvika Sjukhus^1^ |  |  |
|  |  | Avesta Lasarett^2^ |  |  |
|  |  | Bollnäs Sjukhus^1^ |  |  |
|  |  | Gällivare Sjukhus^1^ |  |  |
|  |  | Hudiksvalls Sjukhus^1^ |  |  |
|  |  | Hässleholms sjukhus^1^ |  |  |
|  |  | Höglandssjukhuset Eksjö^1^ |  |  |
|  |  | Kalix Sjukhus^1^ |  | ***i*** |
|  |  | Karlskoga lasarett^1^ |  |  |
|  |  | Kiruna Lasarett^2^ |  |  |
|  |  | Kullbergska sjukhuset^1^ |  |  |
|  |  | Kungälvs sjukhus^1^ |  |  |
|  |  | Lasarettet Enköping^1^ |  |  |
|  |  | Lasarettet i Landskrona^1,2^ |  | ***i*** |
|  |  | Lasarettet i Motala^1^ |  |  |
|  |  | Lasarettet i Trelleborg^1^ |  |  |
|  |  | Lasarettet i Ystad^1^ |  |  |
|  |  | Lasarettet Ljungby^1^ |  |  |
|  |  | Lindesbergs lasarett^1^ |  | ***i*** |
|  |  | Lycksele lasarett^1^ |  |  |
|  |  | Mora Lasarett^1^ |  |  |
|  |  | Norra Älvsborgs Länssjukhus och Uddevalla Sjukhus^1^↓ |  | ***i*** |
|  |  | Norrtälje Sjukhus^1^ |  |  |
|  |  | Nyköpings lasarett^1^ |  |  |
|  |  | Oskarshamns sjukhus^1^ |  |  |
|  |  | Piteå Sjukhus^1^ |  |  |
|  |  | Skellefteå lasarett^1^ |  | ***i*** |
|  |  | Sollefteå Sjukhus^1,2^ |  | ***i*** |
|  |  | Södertälje Sjukhus AB^1^ |  |  |
|  |  | Torsby Sjukhus^1^ |  |  |
|  |  | Visby lasarett^1^ |  |  |
|  |  | Värnamo sjukhus^1^ |  |  |
|  |  | Västerviks sjukhus^1^ |  |  |
|  |  | Västmanlands Sjukhus Köping ^1^ |  |  |
|  |  | Ängelholms sjukhus^1,2^ |  |  |
|  |  | Örnsköldsviks Sjukhus^1^ |  |  |
| Excluded Hospitals |  | Akademikliniken Göteborg |  |  |
|  |  | Akademikliniken Malmö |  |  |
|  |  | Akademikliniken Stockholm |  |  |
|  |  | Aleris Obesitas Skåne |  |  |
|  |  | Aleris specialistvård Sabbatsberg |  |  |
|  |  | Allmänpsykiatrisk vård, vuxna |  |  |
|  |  | Art Clinic Göteborg |  |  |
|  |  | Art Clinic Jönköping |  |  |
|  |  | Art Clinic Uppsala |  |  |
|  |  | Arvidsjaurs sjukhem |  |  |
|  |  | Axlagården Umeå hospice |  |  |
|  |  | Bergslagssjukhuset Mitt Hjärta |  |  |
|  |  | Blackebergs sjukhus^3^ |  |  |
|  |  | Borlänge sjukhus^3^ |  |  |
|  |  | Capio Lundby Närsjukhus |  |  |
|  |  | Capio Movement Halmstad |  |  |
|  |  | Carema Rehabcentrum i Saltsjöbaden |  |  |
|  |  | Carlanderska sjukhuset |  |  |
|  |  | Dalens sjukhus^2^ |  |  |
|  |  | Distriktsläkarvård |  |  |
|  |  | Distriktsläkarvård och Geriatrisk rehabilitering |  |  |
|  |  | Dorotea Sjukstuga |  |  |
|  |  | Elisabethsjukhuset^2^ |  |  |
|  |  | Enskede Årsta Vantörs socialpsykiatriska |  |  |
|  |  | Ersta sjukhus^3^ |  |  |
|  |  | Farsta Årsta Vantörs socialpsykiatriska enhet |  |  |
|  |  | Geriatrisk rehabilitering |  |  |
|  |  | GynStockholm |  |  |
|  |  | Handens Närsjukhus^3^ |  |  |
|  |  | Handens socialpsykiatriska enhet |  |  |
|  |  | Härnösands sjukhus^2^ |  |  |
|  |  | Jakobsbergs sjukhus |  |  |
|  |  | Jokkmokks sjukhem |  |  |
|  |  | Kristinehamns sjukhus |  |  |
|  |  | Lasarettet i Finspång |  |  |
|  |  | Lidingö sjukhus |  |  |
|  |  | Ludvika lasarett^3^ |  |  |
|  |  | Långbro sjukhus |  |  |
|  |  | Löwenströmska sjukhuset |  |  |
|  |  | Malå Sjukstuga |  |  |
|  |  | Maria ungdomsmottagning |  |  |
|  |  | Mariamottagningarna |  |  |
|  |  | Nacka Närsjukhus^3^ |  |  |
|  |  | Nynäshamns sjukhus^3^ |  |  |
|  |  | Närvårdsavdelningen i Uppsala |  |  |
|  |  | Odenplans läkarhus |  |  |
|  |  | Ortho o Spine Center |  |  |
|  |  | Ortopedisk vård |  |  |
|  |  | Ortopedisk vård, Ryggsjukvård, Sjukgymnastikverksamhet |  |  |
|  |  | Pajala sjukhem |  |  |
|  |  | Psykiatriska klinikerna i Växjö |  |  |
|  |  | Regionsjukhuset Karsudden |  |  |
|  |  | Rehab Station Stockholm |  |  |
|  |  | Rehabcenter Sfären - Bräcke diakoni |  |  |
|  |  | Ryggkirurgi Strängnäs |  |  |
|  |  | Ryggkirurgiska kliniken i Strängnäs |  |  |
|  |  | Rättspsykiatriska kliniken i Säter |  |  |
|  |  | Rättspsykiatriska regionkliniken i Vadstena |  |  |
|  |  | Sabbatsbergs sjukhem |  |  |
|  |  | Simrishamns sjukhus |  |  |
|  |  | Sjukhusen i Väster |  |  |
|  |  | Sjukhuset i Säffle |  |  |
|  |  | Sollentuna sjukhus |  |  |
|  |  | Sophiahemmet^2,3^ |  |  |
|  |  | Sorsele Sjukstuga |  |  |
|  |  | Specialist Center Skåne AB |  |  |
|  |  | St Eriks Ögonsjukhus^2^ |  |  |
|  |  | Stockholms sjukhem |  |  |
|  |  | Stora Sköndals sjukhus |  |  |
|  |  | Storumans Sjukstuga |  |  |
|  |  | Södermalm Gamla Stan Socialpsykiatriska Enhet |  |  |
|  |  | Tärnaby Sjukstuga |  |  |
|  |  | Victoriakliniken |  |  |
|  |  | Vilhelmina Sjukstuga |  |  |
|  |  | Västmanlands sjukhus Sala^4^ |  |  |
|  |  | Ytterö behandlingshem |  |  |
|  |  | Åsele Sjukstuga |  |  |
|  |  | Öjeby sjukhem |  |  |
|  |  | Överkalix sjukhem |  |  |
|  |  | Övertorneå sjukhem |  |  |

**Table A2.** **Hospital level classification.** ↓↑ indicates changes in classification compared to Ydenius et al. [4]. Full details of the SPOR categorisation are available in Appendix B.

**Legend for classification method:**

1. The hospital is classified according to SPOR.
2. Hospital not included in SPOR; classification confirmed via web search.
3. Hospital not included in SPOR; classification confirmed through verbal communication with hospital directors, heads of operations, trustees, county officials, or healthcare authorities.
4. Hospital not included in SPOR; classification based on email correspondence with hospital directors, heads of operations, or healthcare authorities.

**Notes:**

- Data were retrieved for the period 2008–2021. During this period, Karolinska University Hospital underwent a major reorganisation, resulting in the establishment of New Karolinska Hospital (NKS).
- *Sahlgrenska Universitetssjukhuset* encompasses Sahlgrenska THX, Sahlgrenska Östra, Sahlgrenska Mölndal, and Sahlgrenska ANOPIVA in the registries of the National Board of Health and Welfare.

**Clarifications on specific hospitals:**

- **Blekingesjukhuset** is split into Karlskrona (Level 2) and Karlshamn (Level 3) in SPOR. This distinction is not made in the National Board of Health and Welfare registries; therefore, for consistency with Ydenius et al. [4], it remains classified as Level 2.
- **Hallands Sjukhus Halmstad** includes the hospitals of Varberg and Kungsbacka, which are not separately listed in the National Board registries. It is retained as Level 2 for consistency with Ydenius et al. [4].
- **Skaraborgs Sjukhus** includes Kärnsjukhuset Skövde (Level 2 in SPOR), Lidköping Hospital (Level 4 in SPOR), and Falköping Hospital (Level 4 in SPOR). As the National Board registers them collectively under Skaraborgs Sjukhus, they are collectively retained as Level 2 in line with Ydenius et al. [4].
- **Lasarettet Landskrona** is defined as Level 8 in SPOR due to uncertain surgical volume. A web search confirmed the presence of an emergency department, but the hospital does not meet anaesthesia requirements for Level 1 or 2; it is therefore classified as Level 3 in this study.
- **Uddevalla Hospital and Norra Älvsborgs Länssjukhus (NÄL)** are grouped together in the National Board’s registries. In SPOR, Uddevalla was downgraded to Level 4 following the transfer of emergency medicine to NÄL in 2008–2009, while emergency orthopaedics remained at Uddevalla until 2015. NÄL is also downgraded to Level 3 in SPOR. The combined entity is classified as Level 3 in this study.
- **Sollefteå Sjukhus**, **Kalix Sjukhus**, and **Lindesbergs Lasarett** are each listed as Level 4 in SPOR due to the absence of maternal care. However, as maternity services were not relevant to this study, all three hospitals are classified as Level 3.


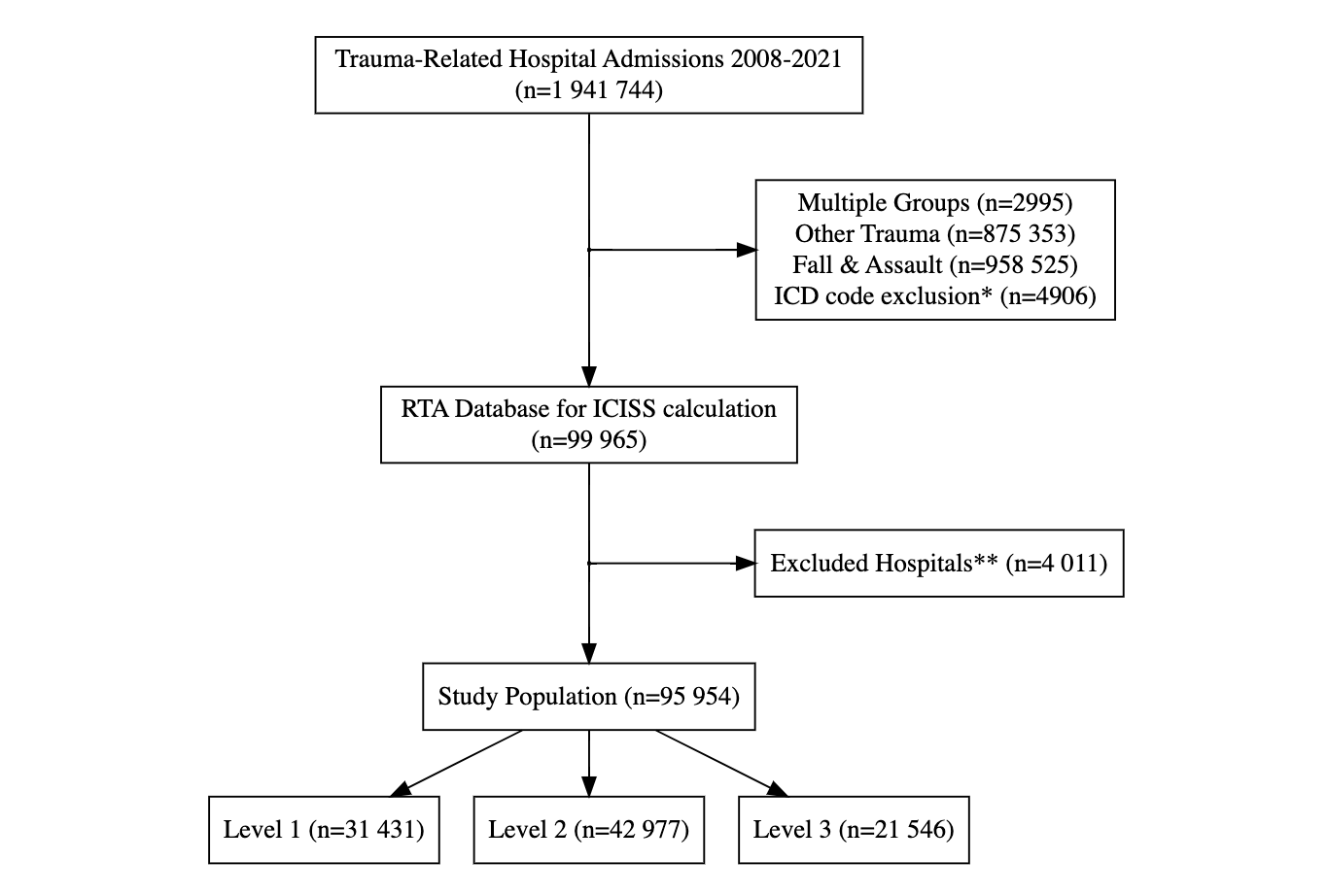


**Figure A1. Flowchart for ICISS calculation**. ‘Other trauma’ refers to injuries that do not fall under the categories of fall, assault, or traffic-related trauma. ‘Multiple groups’ are defined as hospital admissions with overlapping E-codes across the categories of fall, assault, or traffic. Falls and assaults were excluded before constructing the traffic-related trauma database. *To ensure the use of ICD codes focused exclusively on anatomical injury, codes within the ICD-10 trauma chapter (S00–T79) that did not meet this criterion were excluded. **Hospitals that did not meet the inclusion criteria were excluded. See Table A2 in the Appendix for further details.

**
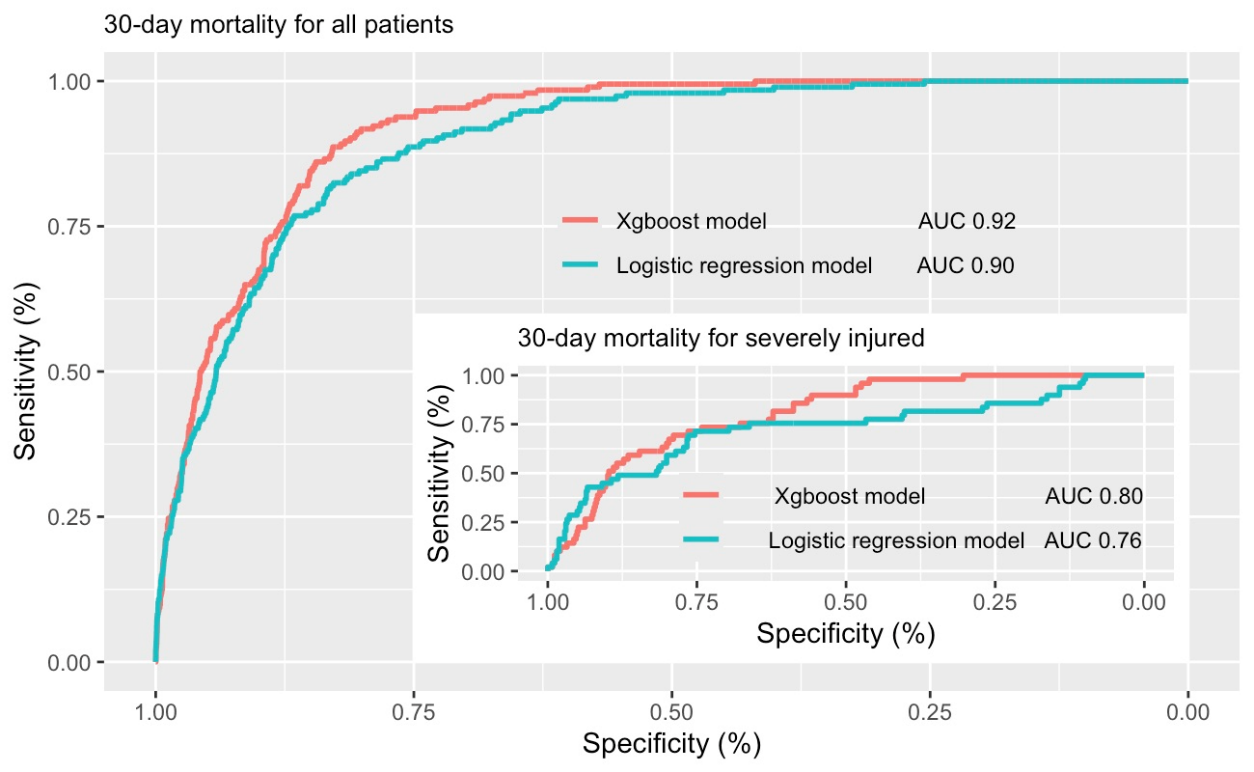
Figure A2. Receiver operating characteristic (ROC) curves and corresponding areas under the curve (AUC) for 30-day mortality prediction**, comparing the logistic regression model and the machine learning model.
